# Synthesis and Luminescent Properties of Carbon Nanodots Dispersed in Nanostructured Silicas

**DOI:** 10.3390/nano11123267

**Published:** 2021-12-01

**Authors:** Andrii Vasin, Dmytro Kysil, Andriy Rusavsky, Oksana Isaieva, Alexander Zaderko, Alexei Nazarov, Volodymyr Lysenko

**Affiliations:** 1 Lashkaryov Institute of Semiconductor Physics, NAS of Ukraine, 03028 Kyiv, Ukraine; kdmitr93@gmail.com (D.K.); rusavsky@yahoo.com (A.R.); oksanka.isayeva@gmail.com (O.I.); nazarov51@yahoo.com (A.N.); lysenko@isp.kiev.ua (V.L.); 2 Department of Applied Physics, National Technical University of Ukraine “Igor Sikorsky Kyiv Polytechnic Institute”, 03056 Kyiv, Ukraine; 3 Institute of High Technologies, Taras Shevchenko National University of Kyiv, 01033 Kyiv, Ukraine; indeo1016@gmail.com; 4 Department of General Physics and Solid State Physics, National Technical University of Ukraine “Igor Sikorsky Kyiv Polytechnic Institute”, 03056 Kyiv, Ukraine

**Keywords:** carbon nanodots, porous silica, fumed silica, SiOC thin films

## Abstract

Luminescent carbon nanoparticles are a relatively new class of luminescent materials that have attracted the increasing interest of chemists, physicists, biologists and engineers. The present review has a particular focus on the synthesis and luminescent properties of carbon nanoparticles dispersed inside nanostructured silica of different natures: oxidized porous silicon, amorphous thin films, nanopowders, and nanoporous sol–gel-derived ceramics. The correlations of processing conditions with emission/excitation spectral properties, relaxation kinetics, and photoluminescence photodegradation behaviors are analyzed. Following the evolution of the photoluminescence (PL) through the “from-bottom-to-up” synthesis procedure, the transformation of molecular-like ultraviolet emission of organic precursor into visible emission of carbon nanoparticles is demonstrated. At the end of the review, a novel method for the synthesis of luminescent and transparent composites, in form of nanoporous silica filled with luminescent carbon nanodots, is presented. A prototype of white light emitting devices, constructed on the basis of such luminophores and violet light emitting diodes, is demonstrated.

## 1. Introduction

Luminescent materials take an important place in many areas of human activity and in daily life, including various optoelectronic devices, artificial lighting, and visualization systems. In recent decades, new luminescent materials based on nanosized particles have been actively developed. The luminescent properties of such materials have many advantages compared to those of the bulk material, due to their large specific surface areas and quantum confinement effects [1,2,3,4,5,6,7,8,9,10,11,12,13,14,15,16]. Among the newest nanostructured luminescent materials, carbon nanoparticles, commonly known as carbon nanodots (CNDs), are of special interest. The term CNDs is commonly used to refer to carbonaceous particles with a size of less than 10 nm. However, the structure and morphology of these particles can be quite diverse. They can include nanoparticles of diamond and graphite, as well as amorphous nanoparticles with a diamond-like, polymer-like, or graphite-like structure [10,11,13,17,18,19]. Nanoflakes of graphene and graphene oxide are also often referred to as CNDs [10,11,17,19].

Unlike the well-defined carbon atom arrangements in fullerenes, nanotubes, graphenes, and diamond nanoparticles, the structural configurations of CNDs are quite uncertain and cannot be definitely specified. Usually, they are represented as combinations of molecular-like polycyclic clusters with sp^2^-coordinated carbon atoms, as well as aggregates of these clusters and amorphous sp^3^-coordinated networks. Due to their lack of a clear shape, CNDs have a developed defective surface with a variety of functional groups and dangling bonds on it. The variety of properties of luminescent centers in CNDs determines the characteristic features of the photoluminescence (PL) spectra of this material, such as a broad emission band, as well as the dependence of the emission spectrum on the wavelength of the exciting radiation, i.e., the so called “excitation dependent photoluminescence” [10,11,17,20,21].

Unlike luminescent semiconductor quantum dots, CNDs are quite a friendly material for the human body; therefore, this material is under intensive development as an effective nanoscale luminescent label for the visualization of biological objects in biomedical experiments. The excitation-dependent emission of CNDs used as fluorescent nanomarkers in biological experiments makes it possible to identify a useful CND signal from the interfering fluorescence of cells or contaminants. It should be noted, though, that excitation dependence is not a “universal” feature and, in some research studies, it has not been observed [17,22,23,24]. Compared to semiconductor nanoparticles and organic fluorophores, CNDs are not subject to the effect of blinking (fluorescence intermittency) [17,19,25]. Due to their relatively high synthesis temperature, CNDs are stable in the temperature range of biomedical experiments and can be stored for a long time at room temperature (without refrigeration). They are also stable in a wide range of pH environments (high ion strength) [26,27]. At present, CNDs can be considered as a separate segment of luminescent materials for the visualization of biological objects [28].

There are many methods of synthesizing CNDs. They can, in general, be classified into two types: (1) “from-top-to-bottom” methods, i.e., the fragmentation of bulk material into nanoparticles, and (2) “from-bottom-to-top” methods, i.e., synthesis from organic molecular precursors along with surface fictionalization and passivation [11,17]. The “from-bottom-to-top” methods have key advantages over the “from-top-to-bottom” methods, including being more environmentally friendly, less time-consuming, and allowing for the easy modification of the surface state and composition of the CNDs [17]. A huge number of the processes related to the “from-bottom-to-top” concept and corresponding carbon precursors have been reported. The impressive variety of methods and materials indicates that the formation of luminescent CNDs over the course of spontaneous carbon precipitation is a rather general property.

Most of the “from-bottom-to-top” methods are based on liquid-phase processing. Liquid-phase synthesis makes it possible to obtain dispersions of CNDs with a high quantum yield (QY) of PL. Many researchers have been reported QYs of higher than 60% [29,30,31,32], which are comparable to the QY of modern commercial phosphors based on semiconductor nanoparticles and rare earth metals. As noted above, a broad spectrum of light emission in the visible spectral range is an inherent feature of PL in CNDs. A high QY and a broad emission spectrum in the visible spectral range logically suggests the use of this material as a source of phosphor in artificial lighting devices. However, for optoelectronic applications, the material must be in a condensed state. Unfortunately, CNDs tend to aggregate when being extracted from liquid dispersion, resulting in strong decrease in the QY. The successful application of CNDs in optoelectronics demands a combination of the following: (1) the synthesis of CNDs with a high QYs and suitable emission/excitation spectra; and (2) the effective dispersion of individual nanoemitters inside of optically transparent solid-state matrices.

At the first glance, the most suitable candidate for a matrix is a polymeric material. Indeed, polymers have a low refractive index (that is favorable for luminophor matrices) and, at present, there is a huge range of transparent polymers of various natures and with various properties. Polymers are very convenient in terms of machining and producing components of the desired shape and size. However, the process of dispersing nanoparticles in a polymer faces the problem of high viscosity of the matrix material, epoxy resin, or polymer melt. Homogeneous dispersion at the level of individual nanoparticles in such a medium is quite problematic. Moreover, polymers tend to degrade under conditions of elevated temperature and illumination caused by high-energy photons that are emitted by excitation sources.

Another highly suitable material is silicon oxide. It is a stable, optically transparent, and well-studied material with a low refractive index. For the homogeneous dispersion of the proper amounts of CNDs inside silica, the material should be composed of nanostructures with large specific surface areas in order to minimize CNDs’ aggregation. It is curious that the broad-band visible photoluminescence of carbon-incorporated silica was repeatedly reported on since long before the first publications on luminescent CNDs in 2004 [33,34,35,36]. Despite some of the authors having ascribed this PL to carbon clusters [33,35], there was no common consensus on the nature of this phenomena for a long time. Nowadays, many of those results can be revised and interpreted more correctly in terms of luminescent carbon nanoclusters and/or nanoparticles. Carbon is insoluble in silicon oxide and, therefore, tends towards segregation [37], in addition to interface, or to the formation of carbon nanoclusters and nanoparticles on the silica surface. At present, the interest in silica nanoparticles as a carrier of luminescent CNDs is growing, particularly in the field of submicron/nanoscale biological object imaging [38] as well as in optoelectronic applications [39,40].

A lot of silica based nanostructured materials with high specific surface areas have been developed and are already commercially available. In recent years, a significant amount of research activity has been focused on the development of nanocomposites of CNDs/mesoporous silica nanoparticles (MSNs) for biomedical applications. MSNs are widely used as drug carriers for the targeted delivery of therapeutic agents to the selected points of tissues [41]. The unique mesoporous structure of silica facilitates the effective loading of drugs and their subsequent controlled release at the target site. Luminescent CNDs can be attached to MSNs to provide visual control of the MSNs’ locations [42,43,44]. Silica nanospheres (SNSs) can also be used for the effective development of the stability and PL efficiency of CNDs immobilized either on the surface of SNSs or inside them [45,46,47]. It has been demonstrated that such approaches improve the stability and PL brightness through the prevention of external quenching.

The PL decay time in CNDs with silica capsulation layers was found to be strongly dependent on changes in the environment from nanoseconds (fluorescence) in air to seconds (phosphorescence) in water. It was suggested that such effects be used for multiplex devices [48]. An effective level of phosphorescence, with the lifetime and phosphorescence quantum yield of the CNDs being 1.86 s and 11.6%, was found in CNDs with silica capsulation layers. Such hybrid nanoparticles are adequately soluble in water and their effectiveness in in vivo/vitro afterglow imaging was also demonstrated [49].

Another interesting effect found in CNDs/silica nanoparticles is the conversion of the excitation-dependent PL mechanism that inherent to pure CNDs into the excitation-independent mechanism found in CNDs grown inside silica nanoparticles. Moreover, the excitation dependence of PL can be fully recovered through the removal of the silica [50]. A non-linear optical response of CND/silica glass nanocomposite was found, and it was suggested that this be used in optical limiters to protect sensitive instruments and human eyes from the damage caused by high-powered lasers [51]. The use of a CNDs/silica hybrid photocatalyst was suggested for the selective utilization of CO_2_ [52].

The present report involves a critical review of the research activity related to the development of luminescent of SiO_2_:C nanocomposites in the form of CNDs dispersed in nanostructured silica with high specific surface areas, such as oxidized porous silicon, amorphous thin films deposited by magnetron sputtering, fumed silica nanopowder, and sol–gel-derived nanoporous silica. The review is limited by the earlier results of our research group, taking into account the constant progress in knowledge and understanding of the materials and related phenomena. First, the SiOC thin layers in the form of carbon-incorporated oxidized porous silicon and amorphous a-SiOC:H thin films, deposited by magnetron sputtering, are considered. Then, the luminescent properties of carbonized fumed silica nanopowders are summarized. Furthermore, at the end of the report, an effective method for the formation of a luminescent nanoporous SiO_2_:C composite, using a combination of liquid-phase CND synthesis and an optically transparent sol–gel-derived nanoporous SiO_2_ template, is demonstrated.

## 2. Por-SiO_2_:C Thin Layers and a-SiOxC:H Thin Films

### 2.1. Oxidized Porous Si:C

**Figure 1:**
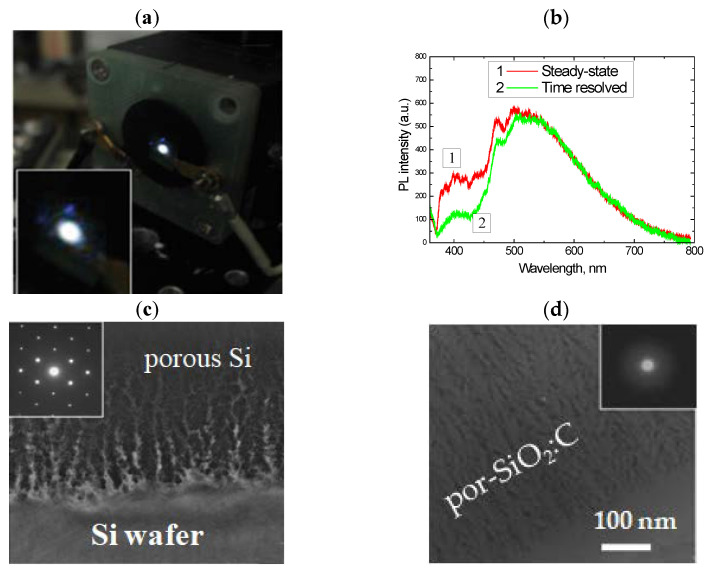
(**a**) Emission spot of por-SiO_2_:C layer under 351 nm excitation (Ar^+^ laser); (**b**) PL spectra of por-SiO_2_:C layer measured in steady-state mode and in time-resolved mode; (**c**,**d**) TEM image of morphology of the carbonized porous silicon layer before and after wet oxidation. Insets in (**c**) and (**b**) represent electron diffraction patterns.

Thin layers of porous SIO_2_:C were formed on the silicon wafer using the following sequence of procedures: electrochemical etching of the Si wafer (formation of porous Si layer) -> carbonization in the flow of acetylene at a high temperature (formation of porous Si:C layer) -> selective oxidation of the porous Si network using water vapor (formation of porous SiO_2_:C layer) [53]. The specific surface area of the as-prepared porous silicon layer was 200–300 m^2^/g [54], and this sample exhibited red PL, which is typical for such kinds of materials. The carbonization procedure led to the quenching of the red emission and no PL was observed after this step. After the wet oxidation of the carbonized layer, a strong visible PL, which could be observed by the eye as a white light (Figure 1a,b), was observed under 351 nm excitation.

The electron diffraction pattern of the samples subjected to wet oxidation at 800 °C showed diffused halos, indicating the full conversion of the crystalline porous Si into amorphous silicon oxide. However, the morphology of the layer remained porous after oxidation treatment (Figure 1c,d). The oxidation rate of nanostructured silicon in water vapor at such temperatures was high, while the pyrolytical carbon on the silicon surface was quite inert to the water molecules. Therefore, the oxidation of carbonized porous silicon resulted in the formation of a porous silica network filled with carbon nanoprecipitates located on the silica surface and/or encapsulated in the silica.

The spectral properties and intensity of PL in the por-SiO_2_:C layers strongly depended on the synthesis conditions. A common feature of light emission is a broad spectral distribution spread from near UV to near IR, with a maximum-intensity variable from blue to green, depending on the synthesis procedure [54]. Time-resolved PL measurements using an N_2_ laser (pulse duration 10 ns) and a stroboscopic registration system (cutting PL signal within 0.1 ns gate and with 0.7–1.0 ns delay from the start of the laser pulsing) demonstrated that such a broad emission band is not homogeneous, but rather, is composed of a “blue” component with a slow response and a basic “green” band with a faster response (Figure 1b).

**Figure 2:**
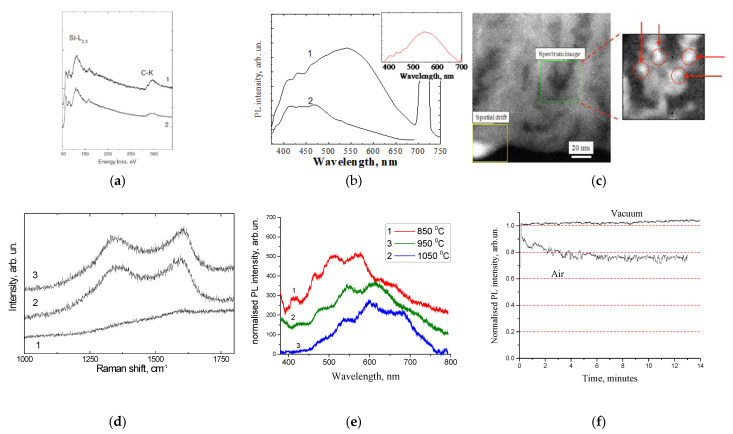
(**a**) Electron energy loss spectroscopy and (**b**) PL spectra of por-SiO_2_:C layer before and after annealing in oxygen at 600 °C (reprinted from ref. [55]); (**c**) HRSTEN/EELS image of distribution of carbon inside pore (reprinted from ref. [56]); (**d**) Raman scattering spectra of porous silicon carbonized at different temperature: 850 °C (1), 950 °C (2), 1050 °C (3) (reprinted from ref. [57]); (**e**) PL spectra of por-SiO_2_:C samples synthesized using different carbonization temperatures; (**f**) evolution of PL intensity in por-SiO_2_:C in time.

The fast component was attributed to the emission centers associated with the carbon that was incorporated into the porous silica layer, and the slow one was attributed to the triplete states of the point defects in the silicon oxide matrix. This assumption was approved by evolution of the PL after annealing in oxygen at 600 °C [55]. After the removal of carbon from the porous layer (Figure 2a), the low-energy PL band was quenched and only the blue component remained detectable (Figure 2b). The inset in the figure shows the result of subtracting the spectrum “after annealing” from the spectrum “before annealing”. This spectrum can be identified as “carbon-related”.

The analysis of the distribution of carbon in the porous layers, using high-resolution scanning transmission electron microscopy (HRSTEM) in combination with energy filtered electron energy loss spectroscopy (Figure 2c), showed that this distribution was not uniform, and that it was characterized by evident precipitates of a few nanometers in size [56].

The effect of the amount of carbon in the porous layer was investigated using samples that were synthesized using different carbonization temperatures [57]. The Raman scattering spectra indicated that the increasing of the carbonization temperature resulted in the growth of the amount of carbon inside the porous layer (Figure 2d), which was accompanied by red shifting of the carbon-related emission (Figure 2e). This spectral shift was preliminary assigned to the growth of the size of the carbon precipitates. As will be shown in the next sections, this effect was quite common for all kinds of luminescent CNDs. The periodic features on the PL spectra (Figure 2e) were caused by the interference of the emission light in the thin porous layers.

The analysis of the time-dependent evolution of the PL intensity under the UV radiation showed significant photo-bleaching in the por-SiO_2_:C layers [57]. It is important to note that photo-bleaching was observed only under atmospheric conditions, while under vacuum conditions, the dynamics of changes in the PL intensity were the opposite, i.e., a slight increase in the PL intensity was observed during the period of the experiment (Figure 2f). Such a behavior implies that the mechanism of photo-bleaching in the por-SiO_2_:C layers was similar to that of the organic fluorophores, i.e., light stimulated the oxidation of the emitters via the oxygen contents in the air.

### 2.2. Luminescent a-SiOC:H Thin Films

**Figure 3:**
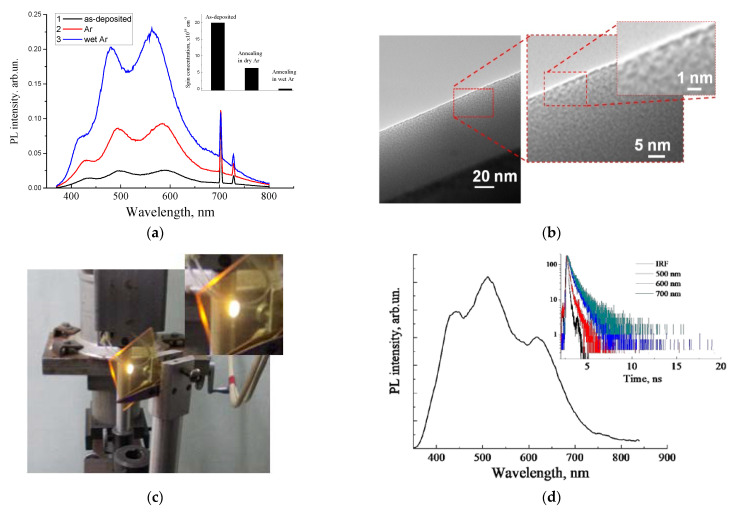
(**a**) PL spectra of the as-deposited and annealed carbon-rich a-SiC:H thin film (inset shows the corresponding evolutions of concentrations of carbon-related paramagnetic centers); (**b**) high-resolution transmission electron microscopy image of the nanoscale morphology of the oxidized a-SiC:H thin film;(**c**) photo image of the emission spot on the as-deposited a-SiOC:H film (excitation by 408 nm), deposited on the glass substrate; (**d**) corresponding PL spectrum and decay curves, measured at different emission wavelengths.

A similar methodological concept to the oxidation of carbonized porous silicon layers was adopted for the amorphous silicon-carbon alloy thin films deposited using the magnetron sputtering technique. It is well known that crystalline silicon carbide (SiC) is a chemically inert and corrosion-resistant material. However, low density amorphous hydrogenated silicon-carbon alloy films (a-Si_1−x_C_x_:H) deposited at low substrate temperatures can be easily oxidized by oxygen or water vapor at temperatures as low as 700 °C, or even lower [58,59,60]. As was found earlier, a-Si_1−x_C_x_:H enriched by carbon (x > 0.5) exhibiteda higher oxidation rate than that of the stochiometric films (x = 0.5). Figure 3a represents the evolution of the PL of the as-deposited carbon-rich a-SiC:H thin films after annealing in dry Ar and wet Ar at 450 °C for 30 min. The PL spectrum of the as-deposited film is a broad emission band covering the entire visible range.

Periodic modulation occurred due to the effect of interference [57,59,61], which was similar to that observed in por-SiO_2_:C thin layers. It can be seen that the PL intensity significantly increased after low-temperature annealing in an inert medium, which was associated with the passivation of paramagnetic dangling bonds by hydrogen, which were released from metastable bound states at low temperature, or with oxidation with residual oxygen [59,62].

A further increase in the PL intensity was observed after oxidation with water vapor; that was correlated with the further decreasing of the concentration of the paramagnetic centers (inset in Figure 3d). FTIR measurements revealed a partial oxidation of the amorphous SiC network. HRTEM examination of the carbon-rich film showed an inhomogeneous density distribution (Figure 3b), which can be interpreted as the nanoporous morphology of the amorphous SiC/SiO_x_:C film.

An alternative process of formation of luminescent silica-carbon layers without the oxidation annealing procedure, performedthrough the deposition of carbon-enriched a-SiOC:H films using excess carbon precursor (methane) in the magnetron deposition process, was suggested [63]. Strong photoluminescence was observed only in a-SiOC:H films with high carbon incorporation (Figure 3c). The emission bands of such a-SiOC:H films were quite similar to those observed in the por-SiO_2_:C layers and oxidized carbon-rich a-SiC:H thin films, with similar regular interference features in the spectrum distribution (Figure 3d).

It should be noted that the optical interference of emission light inside thin layers is a serious obstacle to the adequate analysis of the spectral properties of emission light. From this point of view, materials in bulk form are preferable, and these materials are discussed in the next sections.

## 3. Luminescent Nanopowder: Carbonized Fumed Silica

**Figure 4:**
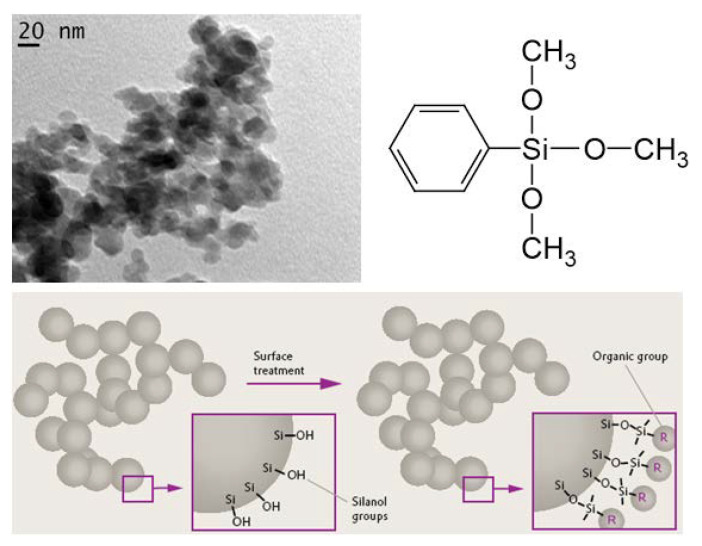
Typical TEM image of the fumed silica used in the research; molecular structure of phenyltrimethoxysilane; and illustration of the chemistry of fumed silica surface modification.

A recent review on synthetic efforts aimed at obtaining CNDs with strong emission in the broad spectral region, particularly with high QY in red, and the fabrication procedures intended to produce CND-based composites with stable optical responses, can be found in [64]. In terms of optoelectronic applications, the porous SiO_2_:C thin layers grown on Si wafer, as well as carbon-rich a-SiOC:H thin films, seem not to be sufficiently suitable when taking into account the related technological complexities. For the further development of luminescent SiO_2_:C materials towards practical applications, it was suggested to implement fumed silica nanopowder as a morphological matrix for luminescent CNDs. The morphology of fumed silica nanopowder with a specific surface area of 295 m^2^/g was represented by silicon oxide particles of 10–20 nm in size, which were fused and formed agglomerates of tens/hundreds of nanometers in size (Figure 4).

The surfaces of the nanoparticles were coated with hydroxyl groups, which were used to modify the surface by means of substitution reactions. The main idea of the present method was the immobilization of organic and/or organosilicon functional groups on the surface of silicon oxide by means of covalent bonds, followed by their pyrolysis to form carbon precipitates. From the point of view of chemistry, the most effective immobilization is via the substitution reaction of hydroxyl groups with the silyl groups of organosilicon compounds such as methoxysilane and phenyltrimethoxysilane (PhTMS) [65,66,67]. It was found that PhTMS appeared to be more effective in the formation of luminescence materials, presumably due to the presence of a phenyl group, which effectively participated as a “building block” in the process of carbon precipitation.

**Figure 5:**
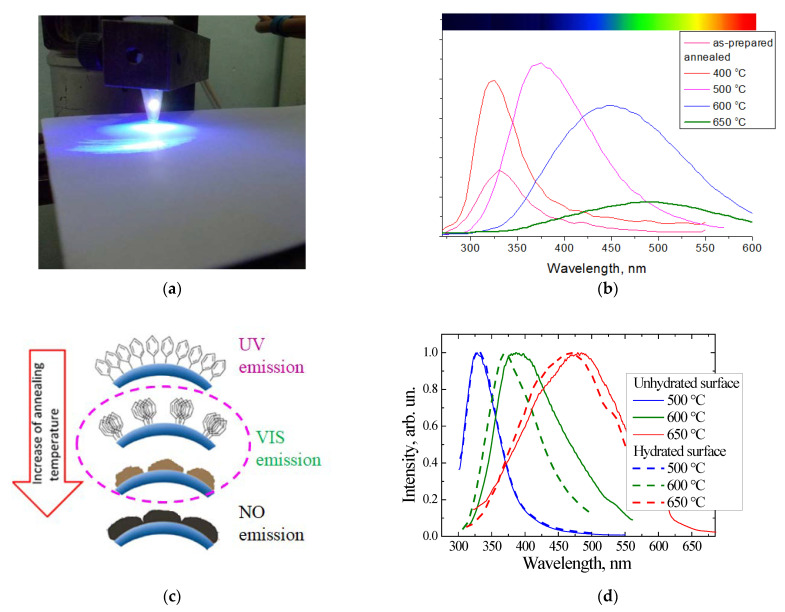
(**a**)A typical image of the emission of SiO_2_:C nanopowder under LED excitation (λ_ex_ = 408 nm, power = 40 mW); (**b**) PL spectra of “phenyl-silica” after annealing at different temperatures (excitation 250 nm); (**c**) illustration of the processes of pyrolytical precipitation of carbon on the silica surface; (**d**) emission spectra of annealed phenyl-silicas with and without surface hydration pretreatment.

The evolution of the UV emission of phenyl groups into the visible emission of CNDs dispersed in fumed silica was studied via the variation in the concentration of PhTMS and in the temperature of pyrolysis [66,67]. The photo in Figure 5a illustrates the emission of a SiO_2_:C nanopowder under the excitation of a 408 nm 40 mW LED at room lighting. The emission has a very broad spectral distribution, which is visually perceived as white light.

The main synthesis parameters that determined the intensity and spectral distribution of radiation were the nature and amount of the carbon precursor incorporated in the matrix, the temperature and duration of pyrolysis, and surface pretreatment of fumed silica. Figure 5b shows the evolution of the PL spectra of nanopowders synthesized using PhTMS as a carbon precursor (“phenyl-silica”) with a pyrolysis temperature in the range of 400 to 650 °C at a fixed pyrolysis time of 30 min. It can be seen that the increasingof the pyrolysis temperature led to a gradual low energy shift of the PL band. At annealing temperatures above 500 °C, this shift was accompanied by a decreasing of the integral intensity.

The UV PL band of the as-prepared “phenyl-silica” samples was ascribed to the emission of excimers of phenyl groups bonded to silicon atoms. At low temperature annealing (400 °C), the phenyl groups migrated over the surface and formed domains with increased concentration (Figure 5c), thus increasing the intensity of the excimer emission. With a further increasingof the temperature, the process of structural rearrangement of the organic component began with the formation of carbon precipitates, which resulted in the shifting of the PL band in the visible spectral range [66]. At a pyrolysis temperature of 600 °C and above, the long-wavelength shift was accompanied by the quenching of the emission intensity. Red shifting was also caused by increasing of the amount of PhTMS carbon precursor [67] and was initiated to increase the size of the carbon nanoprecipitates. The higher the annealing temperature and the concentration of carbon precursor, the more intense the clusterization process; this was due to the intensification of the decomposition of organic groups and of surface diffusion processes.

It was also found that the surface mobility of carbon played an important role in the precipitation process. This was the case because the surface pretreatment of the fumed silica by means of hydration [68], in order to improve the immobilization of the phenyl groups on the silica’s surface, was found to prevent carbon precipitation (Figure 5d).

**Figure 6:**
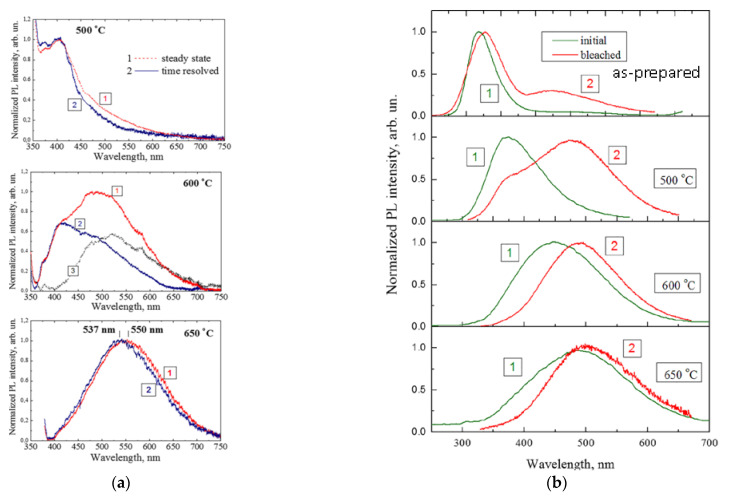
(**a**) Comparison of steady state and time resolved PL spectra of “phenyl-silica” annealed at different temperatures; (**b**) the effect of photo-bleaching on the spectral evolution of PL of as-prepared and annealed phenyl-silicas (reprinted from ref. [66]).

In order to examine the homogeneity of the emission spectra of SiO_2_:C nanopowder with respect to the PL response, the samples were examined by time-resolved PL spectroscopy, under excitation with a 337 nm N_2_ pulse laser [66], by means of comparison the with por-SiO_2_:C described in the previous section. It can be seen from Figure 6a that the steady-state and time-resolved spectra of the samples annealed at 500 °C and 650 °C were almost identical, indicating good homogeneity of the ensemble of emitters with respect to the PL response time. In contrast, PL band of the sample annealed at 600 °C exhibited strong inhomogeneity with almost equal contribution from the “fast” and “slow” centers. Subtracting the time-resolved spectrum from the steady-state yielded a spectral distribution similar to that of the sample annealed at 650 °C (spectrum 3 in Figure 6a). From such comparisons, it can be suggested that the emission band of this sample was composed of at list two components corresponding to emission centers that had a structural nature similar to those that were dominant in the samples annealed at 500 °C and 650 °C. In summary, such experiments demonstrated the multiband nature of PL in SIO_2_:C nanopowders.

The multiband nature of the light emission of SiO_2_:C nanopowder could be effectively “visualized” in the bleaching evolution of PL. It was found that the SiO_2_:C samples in atmospheric air exhibited obvious time-dependent PL extinction and spectral evolution, while measurements in the flow of pure nitrogen did not reveal spectra changes under UV radiation [66]. Figure 6b shows the normalized PL spectra measured in nitrogen flow and in air. From the comparison of the spectra, it could be concluded that the high-energy PL emission is more sensitive to bleaching effects. Such a conclusion was confirmed by the experiments involving the direct examination of bleaching dynamics using the strong UV-radiation of an Hg-lamp as a radiation source (supplementary information for [66]): Exposure of the samples to UV radiation for 12 min resulted in the decreasing of the 440 nm emission intensity by about 48%, while the 530 nm emission was reduced by 23%. This observation illustrates that the UV-emitting centers in SiO_2_:C are less stable against photo-bleaching, and this observation can be assigned to the more “organic nature” of these centers in contrast to the “inorganic nature” of long wave-length emitting centers.

The evolution of the integral PL intensity in a-SiO_2_:C sample in air and in vacuum was studied in [67]. A typical monotonous reduction in PL intensity was observed in an ambient atmosphere, while in a vacuum, the PL dropped in a step-like manner directly after the vacuum pump was switched on, and no further degradation was observed. After extracting the sample from the vacuum chamber, the emission intensity was recovered within 1–2 days of exposure to the air. The step-like drop in PL intensity observed after air evacuation and the recovery observed after exposure to air indicate the important role of desorption/absorption processes involving some of the air components. These experiments point out the opposite effects of the air: (i) the “destructive” effect of bleaching in air under UV radiation, and (ii) the “constructive” effect of adsorption of some atmospheric components that significantly improves the PL efficiency. At present, it is not specified which of atmospheric components is responsible for the “constructive” effect.

## 4. Sol–Gel-Derived Nanoporous Silica

In the previous section, highly dispersed nanopowders of fumed silica were considered as a nanostructured matrix for pyrolytically synthesized CNDs using PhTMS as a carbon precursor. This method made it possible to follow the evolution of PL from the UV emission from phenyl groups to the broad-band visible PL of carbon nanoprecipitates (CNDs). However, due to the inherent inhomogeneity of its morphology, at a scale of hundreds of nanometers, this material strongly scatters light in the visible spectral range and is not sufficiently suitable as a luminophor. In fact, luminophor-related applications need optically transparent materials. As a further means towards making progress, it was suggested to use transparent nanoporous silicon ceramics synthesized by the sol–gel method. Two types of material, with an average pore size of 7 nm (specific surface area 400 m^2^/g, optically transparent) and 40 nm (specific surface area 90 m^2^/g, optically opaque), were studied. The synthesis procedure and material properties are described in more detail elsewhere [69].

Two methods were implemented for the dispersion of luminescent CNDs inside the porous layer: (1) a “Solid-state process”, which is similar to that described in the previous section; i.e., a successive procedure for the immobilization of phenyl groups on the silica surface with subsequent pyrolysis; (2) a “liquid-phase process”, namely the infiltration of a porous material with water dispersions of luminescent CNDs.

“Solid-state process”. This process is quite similar to that used for the fabrication of carbonized fumed silica (see part 3), i.e., the chemical treatment of porous silicas via the toluene solution of phenyltrimethoxysilane followed by pyrolysis at temperature up to 600 °C, for 30–180 min, in the flow of pure nitrogen. The effect of the temperature and duration of annealing, as well as the effect of the amount of carbon precursor on the spectral evolution of PL, were found to be the same as in case of fumed silica: the increasingof the annealing temperature, annealing duration, and carbon amount resulted in the spectral shift to long wavelengths and the broadening of the emission band [70]. The similar multiband nature of the emission spectrum in the near-UV/VIS spectral range was also demonstrated. Based on the similarity of the PL spectra and their evolution with temperature, we suggest that the structural evolution of phenyl groups in por-SiO_2_wassimilar, i.e., carbon clustering caused by fusing and/or the destruction of phenyl groups resulting in the formation of amorphous carbon nanoprecipitates and molecular-like polycyclic fluorophores [67].

“Liquid phase process”. Usually, for the liquid-phase synthesis of CNDs, a heat treatment (thermolysis) at a temperature close to the boiling point of water is used. Such high temperature processing needs special equipment, such as autoclaves, to maintain a temperature near/above water boiling temperature. As an alternative to that method, it has been suggested that the thermolysys of the carbon precursor not be performed in water, but in a high-boiling-point organic solvent, such as dimethylsulfoxide (DMSO). To simplify the process even more, sucrose was used as a carbon precursor. The boiling point of DMSO is about 189 °C, which is significantly higher than the destruction temperature of sucrose molecules, which occurs at temperatures above 160 °C.

**Figure 7:**
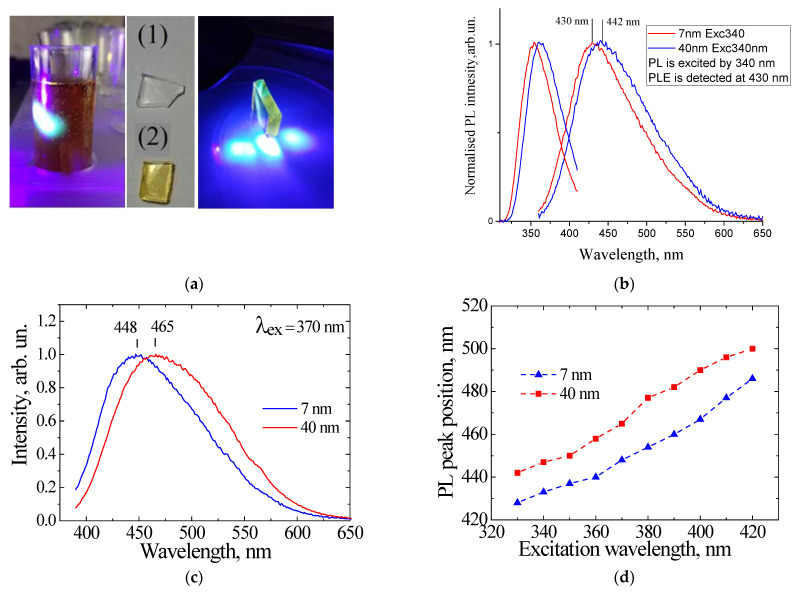
(**a**) Photos of the dispersion of CNDs synthesized from sucrose/DMSO solution, samples of porous silica with 7 nm pore size before (1) and after infiltration (2), and light emission of por-SiO_2_:CNDs under 409 nm LED (right photo); (**b**) emission and excitation PL spectra of CNDs synthesized from sucrose/DMSO solution and extracted into water from porous silica with pore sizes of 7 nm and 40 nm; (**c**) PL spectra of CNDs synthesized from sucrose/DMSO inside pores with pore sizes of 7 nm and 40 nm (reprinted from ref. [72]); (**d**) peak positions of PL of the CDs synthesized using different matrices vs. excitation wavelengths(reprinted from ref. [72]).

Two approaches were implemented for preparing luminescent porous SiO_2_:C samples. First, the thermolysis of sucrose in DMSO solution was performed at temperatures of 170–180 °C, resulting in a dark brown dispersion with strong visible PL (Figure 7a). the infiltration of porous silicon oxide with the luminescent dispersion was carried out by immersing the sample in the dispersion for 24 h, followed by drying. After this procedure, the sample acquired a uniform light brown color and showed intense visible PL (see Figure 7a). To study the effect of the size of CNDs on their PL properties, the porous silica samples were immersed into water for 24 h, resulting in light brown luminescent dispersion. The idea of the experiment was that the average size of the CNDs that penetrated into the porous matrix would be proportional to the pore size of the matrix. Figure 7b shows the emission and PL excitation spectra of CNDs extracted from porous samples with pore sizes of 7 nm and 40 nm. It can be seen that emission and excitation bands were low-energy shifted as the pore size increased from 7 to 40 nm. A similar pore size effect was observed in CNDs synthesized in a porous matrix using citric acid as a carbon precursor [71].

An alternative procedure is the infiltration of porous silicon with sucrose/DMSO solution, followed by thermolysis at 170–180 °C for a few minutes [72]. Figure 7c represents the typical PL spectra of the CNDs synthesized using sucrose (200 mmol/L)/DMSO solution for infiltration followed by thermolysys at 180 °C for 8 min.

The effect of the pore size was quite similar to that observed in CNDs dispersions prepared using the previous method, i.e., a low-energy shifting of the spectrum with the increasing of the pore size.

This shifting of the PL peaks was preserved at different excitation wavelengths (Figure 7d), indicating that the effect originated from the material properties of the CNDs, and was most likely associated with the effect of the size of CNDs. Obviously, the size effect in this case had no relation with the quantum confinement effect that is inherent to semiconductor nanoparticles, and was instead determined by the interplay of excitation/emission and energy transfer between electronic states on the surface and inside the particle.

**Figure 8:**
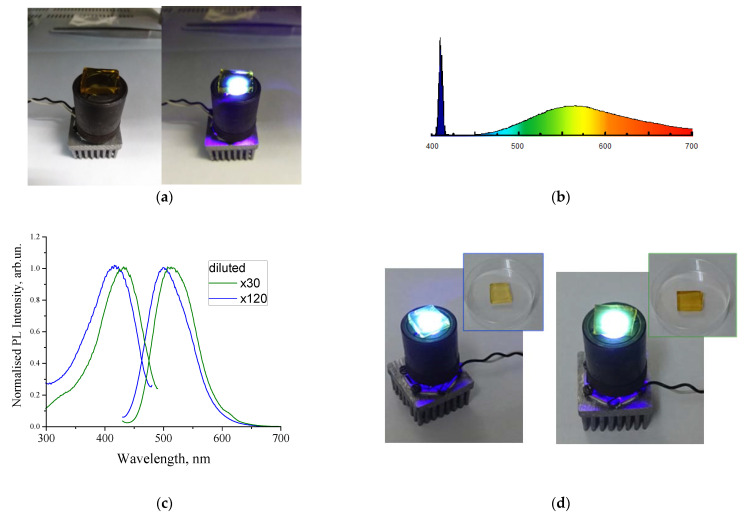
(**a**) Photo images of por-SiO_2_:CNDs sample under incandescent laboratory illumination and under 409 nm LED illumination (reprinted from [72]); (**b**) full emission spectrum of a white-light LED prototype as a combination of a 409 nm semiconductor laser and a por-SiO_2_:CNDs luminophor; (**c**) emission and excitation PL spectra of water dispersions with different concentrations of CNDs; (**d**) photo images of the emission of por-SIO_2_:CNDs infiltrated by dispersions with different concentrations of CNDs.

As it can be seen in Figure 7a, the por-SiO_2_ ceramic material with a pore size of 7 nm was adequately transparent in the visible spectral range. Infiltration by CNDs caused this material to become brownish with its transparency being dependent on the concentration of CNDs. With a proper concentration of CNDs, this por-SiO_2_:CNDs seems to be quite suitable as a luminophor for artificial white light sources. Indeed, among the silica-based matrixes presented in this review, the transparent nanoporous silica with a pore size of 7 nm seems to be most suitable as a container of luminescent CNDs. Figure 8a illustrates a prototype of a white light source that is a combination of a 409 nm LED and por-SiO_2_:CNDs fabricated using CNDs derived from sucrose/DMSO solution [72]. Figure 8b represents the spectrum distribution in a similar light source that, in this case, uses a semiconductor laser for excitation.

Other CNDs have been also used for the demonstration of white light sources. CNDs were synthesized via the solvothermal method using urea, anhydrous citric acid, and 3-(trifluoromethyl) aniline as precursors [73,74].

Figure 8c represents the emission and excitation spectra of dispersions with a concentration that differed by a factor of four. With the increasing of the concentration of CNDs in the dispersion, a noticeable long-wavelength shift was observed. The emission spectra were measured at the same excitation wavelength, so the spectral shift referred solely to the concentration effect. The concentration effect could be associated with both the effect of aggregation in the dispersion and with the effect of the secondary absorption of PL radiation in the phosphor layer. This effect is currently being investigated in more detail. However, it is worth noting that it was possible to tune the spectral properties by changing the concentrations of the emitters. Dispersions of these CNDs were used to infiltrate porous silica samples according to the procedure described in the previous paragraph. The photo in Figure 8d illustrates the emission from CNDs infiltrated by CND dispersion at different concentrations. Regardless of the mechanism of the spectral shift, this observation suggests the possibility of controlling the color tone of white radiation using the concentration of CNDs.

As far as the applications related to artificial lighting are concerned, the problem of the lack of low energy photons in emission of CNDs (orange/red spectral range) should be noted. Commonly, QY is dramatically decreased with the increasing of the emission wavelength. While QYs of over 90% have been widely reported for blue emitting CNDs, the PL efficiencies of green/yellow emitting CNDs were reported to be significantly lower, i.e., in the range of 40–70% [75,76,77], and the QY of red emission rarely exceeded 25% [78,79,80]. Low energy photons are emitted from structural domains with the lowest HOMO-LUMO gap; this is associated with larger sp^2^-conjugated blocks or with their aggregates. However, increases in the size of the conjugated system in an amorphous state, as well as increases in aggregation, are accompanied by the enhancement of nonradiative recombination paths, which results in the reduction in QY. Hence, the development of orange/red emission in CNDs requires the development of an effective means of subtraction of nonradiative recombination paths. To the best of our knowledge, the highest QY of 53% for orange/red emission was reported in [81]. Effective red emission was obtained by increasing of the size of the sp^2^-coordicated structural domains and ensuring proper passivation/functionalization. An alternative method that was capable of increasing the QY of 580 nm emission by up to 46% was suggested, which involved the incorporation of metals in the structure of CNDs [82].

## 5. Summary

Several methods for the dispersion of luminescent CNDs in nanostructured silica matrixes were demonstrated in this study. The photoluminescent properties of the materials were studied in terms of their correlations with synthesis conditions. The evolution of the ultraviolet emission of organic precursors into the visible emission of carbon nanoprecipitates under pyrolysis/thermolysis processes was ascribed to the evolution of the structure of the emitters from well-defined molecular species to molecular-like clusters and, finally, to amorphous carbon precipitates, which are commonly identified as CNDs. It was shown that the visible photoluminescence of CNDs was inhomogeneous and multicomponent, which made it possible to control the spectral properties of broad-band emission by varying the relative content of emitters of different natures. It was also demonstrated that the photo-bleaching effect that is inherent to CNDs can be minimized or even completely avoided via the proper isolation of CNDs from contact with air. Finally, it was demonstrated that CNDs definitely have great potential as luminophores for artificial white light sources. The critical challenges that are still to be met include the avoiding of photobleaching and the development of QY in the red spectral range. Among the materials studied in the present review, the sol–gel-derived nanoporous ceramics with a pore size of 7 nm were found to be the best candidate as a “container” of luminescent CNDs for broad-band visible emission luminophores.
